# A Frequent Serious and Unrecorded Complication of Typhoid Fever

**Published:** 1902-05

**Authors:** W. D. Hoyt

**Affiliations:** Rome, Ga.


### A FREQUENT SERIOUS AND UNRECORDED COMPLICATION OF TYPHOID FEVER.

By W. D. HOYT, M.D.,

Rome, Ga.

For some years past my attention has been turned to the occurrence of a complication of typhoid fever, concerning which I have found no mention elsewhere. The complication is of the nature of a more or less clearly defined chill, with its centripetal and centrifugal stages. It has a periodical tendency. It occurs at any hour of the day, but more frequently, perhaps, near the hour of 10 A.M. than at any other hour. Sometimes I have noted two paroxyisms a day, one in the morning and the other in the afternoon or evening.

The centripetal stage is marked by more or less coldness of the extremities; by an abatement of the temperature; and by a feeling of distress and nervousness. This is followed, after a longer or shorter interval, by the centrifugal stage, characterized by a decided rise in the temperature, by a flushing of the cheeks, and by an increase in the force and frequency of the action of the heart. The rise of temperature is sufficient to make the morning temperature higher than that of the evening, thus constituting an exception to the rule, that in typhoid fever the evening temperature is higher. This complication adds to the gravity and increases the danger of typhoid. The decided rise of temperature occurring day after day lowers the vital forces and lessens the powers of resistance. During the centripetal stage two engorgements and congestions of vital organs are apt to occur, thus gravely complicating the case.

Two years ago I attended a little girl about seven years old, who presented this complication markedly during the first round with the fever. Having a recurrence of the fever, which, in grave cases (as shown by Sir Wm. Gull), is so apt to take place, she presented during this second round a curious periodical engorgement of the lungs. At my morning visit the lungs would be resonant throughout and would present only some coarse mucous valves.

During the morning, engorgement of the lungs would gradually come on with lessened resonance on percussion and with all the symptoms of a severe bronchitis. Treatment only mitigated the attacks, and they did not wholly disappear until the termination of the fever.

In reviewing mentally the cases of fever which I have treated, I feel sure that I lost cases of fever before I made this study, which I would save now; also, that I save now cases which I would have lost. With reference to the frequency of the complication, I would say, roughly, that I find it more or less well defined in fully one-third of the cases I treat. This brings me to the treatment of the complication.

The symptoms being so decidedly those of a chill, and the attacks coming on with regular periodicity, would naturally suggest the use of quinine. I used it, but derived no benefit from it. I confess myself as amongst those who consider quinine not only useless, but positively harmful in typhoid. In searching for something to use in the place of quinine to control this condition, I hit upon opium. From the use of this drug, supplemented by hot appliances to the feet, I have derived so much benefit in these cases that it has not been necessary to search further for remedies. I have used only laudanum, giving it to adults in ten-drop doses, and to children a proportional dose. Often, when I have been able to fix the hour of the attack, I have been able to avert it by giving the laudanum in advance of the hour. Should I not be so fortunate as to avert the attack, I have uniformly succeeded in lightening it. Often after using the remedy a few days the complication ceases to occur. Occasionally I find it necessary to repeat the dose. In exceptional cases it will be necessary to continue the remedy until convalescence is well established, as with the little girl whose case was mentioned. I would emphasize the use of hot applications to the feet. Anything which will keep the blood to the surface or in the extremities, will lessen the centripetal tendency and thus help to avert the paroxysm. I would earnestly invite other observers in different parts of the State to watch carefully for this complication, and to test the efficacy of the advised treatment.

